# HiCUP: pipeline for mapping and processing Hi-C data

**DOI:** 10.12688/f1000research.7334.1

**Published:** 2015-11-20

**Authors:** Steven Wingett, Philip Ewels, Mayra Furlan-Magaril, Takashi Nagano, Stefan Schoenfelder, Peter Fraser, Simon Andrews

**Affiliations:** 1 Bioinformatics Group, The Babraham Institute, Cambridge, CB22 3AT, UK; 2 Nuclear Dynamics Programme, The Babraham Institute, Cambridge, CB22 3AT, UK; 3 Epigenetics Programme, The Babraham Institute, Cambridge, CB22 3AT, UK

**Keywords:** Hi-C, CHi-C, Chromatin, Structure, Epigenetics, Genomics, Bioinformatics, Pipeline

## Abstract

HiCUP is a pipeline for processing sequence data generated by Hi-C and Capture Hi-C (CHi-C) experiments, which are techniques used to investigate three-dimensional genomic organisation. The pipeline maps data to a specified reference genome and removes artefacts that would otherwise hinder subsequent analysis. HiCUP also produces an easy-to-interpret yet detailed quality control (QC) report that assists in refining experimental protocols for future studies. The software is freely available and has already been used for processing Hi-C and CHi-C data in several recently published peer-reviewed studies.

## Introduction

**Figure 1.**
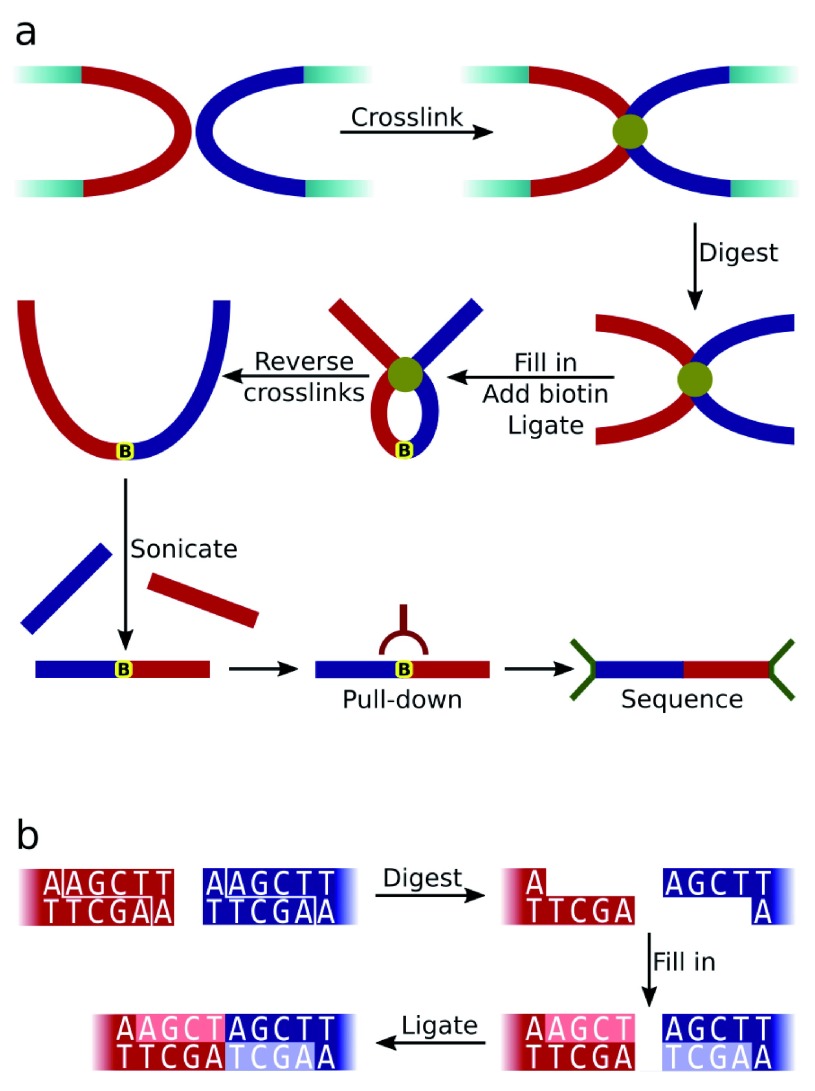
**a**) Diagram summarising the Hi-C experimental protocol. The red and blue rectangles represent cross-linked restriction fragments while the yellow marker shows the position of biotin incorporation. **b**) Generation of the Hi-C ligation junction sequence by successive digestion (with HindIII in this example), fill in and blunt-ended ligation steps. The modified restriction site sequence is not found in the original genomic sequence.

Hi-C is a ligation-based proximity assay utilising the power of massively parallel sequencing to identify three-dimensional genomic interactions ^1^. The method (summarised in Figure 1a) involves fixing chromatin to preserve genomic organisation, followed by restriction enzyme digestion of the DNA. Overhanging single-stranded DNA at the ends of restriction fragments are then filled in with the concomitant incorporation of biotin. Fragments in close spatial proximity are ligated together generating a novel "modified restriction site" sequence (see Figure 1b). Following sonication the sheared ligated DNA fragments are enriched by streptavidin pull-down of the biotin residues, and then are ligated between sequencing adapters. The resulting molecule, termed a di-tag, should comprise two different DNA fragments separated by a modified restriction site. Since these two fragments were positioned close to each other during fixation, by analysing the composition of a population of di-tags generated by a Hi-C experiment it is possible to infer genomic three-dimensional organisation.

A recent variation of the protocol involves enriching Hi-C libraries for di-tags in which one or both reads align to pre-selected regions of a genome ^2– 4^. This Capture Hi-C protocol (CHi-C) is advantageous since Hi-C libraries are extremely complex and even with current high-throughput technologies often only a small proportion of a Hi-C library is sequenced. Selecting for di-tags in this way makes it possible to gain a more complete and higher resolution contact profile for loci of interest.

Reaching valid conclusions regarding genomic interactions requires Hi-C data to be mapped unconventionally (described below) as compared with most paired-end experiments. Following this, artefacts inherent to the Hi-C protocol should be removed. To meet these demands we developed HiCUP, an easy-to-use Hi-C bioinformatics pipeline which has few dependencies and is coded in Perl. HiCUP produces a detailed quality control (QC) report in an interactive HTML format, enabling the user easily to assess the quality of a library and how the experimental protocol may be improved in the future. HiCUP was designed for mapping Hi-C data and removing artefacts. It does not perform the normalisation and statistical tests needed to interpret Hi-C experiments, rather it is intended as the starting point of processing Hi-C datasets and should be used in conjunction with other Hi-C pipelines.

There are numerous packages available to perform different steps in the analysis of Hi-C data. These include Hicpipe ^5^, which is used to generate renormalised contact maps by correcting for pre-determined systematic biases, such as the GC content around the Hi-C ligation junction. Similarly, HiC-Lib ^6^ comprises a set of Python scripts for drawing corrected contact heatmaps, only this technique uses an iterative correction algorithm so that potential biases do not need to be identified prior to processing. Homer ^7^ also creates iteratively corrected heatmaps and includes extra features, such as scripts to identify topologically-associated domains (TADs). In addition, Fit-Hi-C ^8^ takes renormalized data to identify mid-range intra-chromosomal interactions; and GOTHiC ^9^, a Bioconductor package, performs a cumulative binomial test to identify contacts between distal genomic loci that have significantly more reads than expected by chance. Finally, CHiCAGO ^10^ is an open-source R package for interaction detection in CHi-C datasets. Many of the existing tools — including CHiCAGO, Hicpipe, Homer and GOTHiC — can take the output of HiCUP as input for the analyses they perform.

## Methods

### Implementation

HiCUP takes paired-end FASTQ files along with a FASTA reference genome and associated aligner indices and then reports valid di-tags in BAM/SAM format.

**Mapping:** The first stage in the HiCUP pipeline involves truncating reads at the modified restriction site (if present) that separates two DNA fragments. The rationale for this step is similar to that responsible for adapter trimming, namely to remove bases that would otherwise prevent a read mapping to the specified reference genome. After truncation, the resulting trimmed read sent for alignment should, in theory, represent a contiguous genomic sequence derived from a single restriction fragment (i.e. not a hybrid sequence comprising more than one restriction fragment).

HiCUP uses Bowtie ^11^ or Bowtie 2 ^12^ to map Hi-C di-tags, allowing only unique high-quality alignments. Since valid Hi-C reads do not represent one continuous genomic sequence, the pipeline maps forward and reverse reads independently and then re-pairs sequences in which both ends aligned unambiguously to the genome.

**Filtering:** HiCUP removes sequences representing experimental Hi-C artefacts and other uninformative di-tags, which is important since even a small number of invalid di-tags could lead to incorrect conclusions being drawn concerning genomic structure. HiCUP identifies and removes such sequences by positioning putative di-tags on an *in silico* digestion of the reference genome. The processes by which these artefacts are generated experimentally, and how HiCUP identifies them, are described below.

**Figure 2.**
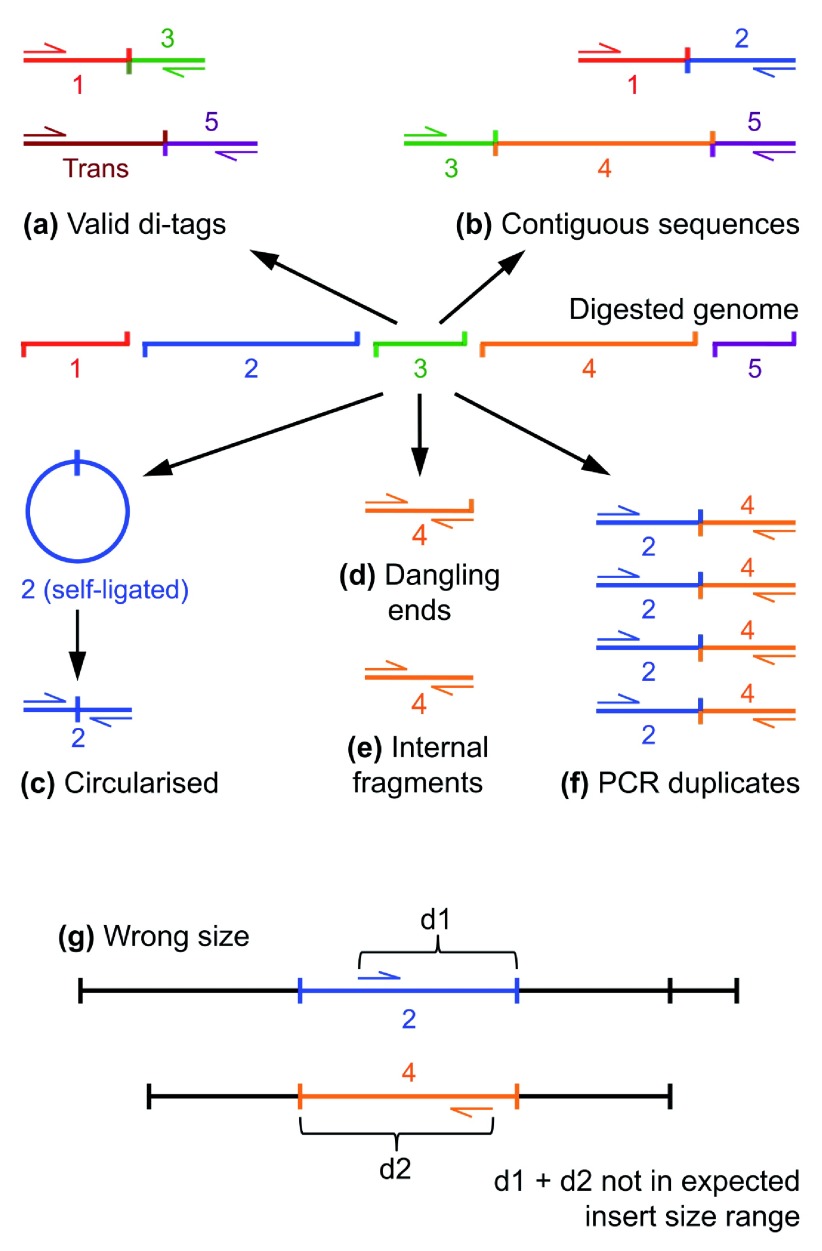
Overview of experimental artefacts generated by the Hi-C experimental protocol. The schematic shows the genome digested into 5 restriction fragments. These fragments may subsequently ligate to each other, or fragments derived from another chromosome, forming valid *cis* or *trans* di-tags respectively ( **a**). In contrast, re-ligation or incomplete digestion leads to the generation of invalid contiguous sequences ( **b**). Another common artefact occurs when the sequenced read-pair maps to a single restriction fragment ( **c**), ( **d**) & ( **e**). Further, PCR may result in a fragment being copied multiple times ( **f**). Di-tags are also rejected when the mapped reads are positioned too far away from the putative restriction enzyme cut-site than allowed by the experimental size-selection step ( **g**).

***Re-ligation of adjacent restriction fragments.*** The Hi-C protocol does not prevent entirely two adjacent restriction fragments re-ligating, but HiCUP discards such di-tags since they provide no useful three-dimensional proximity information. Similarly, multiple fragments could re-ligate forming a contig, but here paired reads will not map to adjacent genomic restriction fragments ( Figure 2b). Furthermore, it is possible that a genome may not be digested completely, also generating molecules spanning adjacent or multiple restriction fragments. Such species should be selected against during biotin pull-down but, nevertheless, are observed in processed datasets.

HiCUP takes the expected size range of the sample (which is predetermined by a di-tag length selection step during library construction) and identifies di-tags in which reads, when mapped to the genome, are separated by a distance falling within the size-selection range. Such di-tags are assumed to comprise a contig spanning several restriction cut sites, and so are discarded.

***Paired reads mapping to the same restriction fragment.*** Read pairs mapping to the same restriction fragment do not describe three-dimensional contacts and therefore should be discarded. A mechanism for generating such aberrant species is when fragments circularise by ligating to themselves and then, following sonication, form linear molecules possessing a valid Hi-C junction. Indeed, inadequate cross-linking can make this problem worse, increasing the frequency with which individual restriction fragments become separated and are therefore unable to ligate to other DNA molecules ^13^. These artefacts are identifiable because the read pairs that map to the same genomic restriction fragment are orientated away from each other when aligned to the reference genome ( Figure 2c). It is also possible for non-ligated DNA fragments to insert between sequencing adapters, despite the protocol being designed to minimise such events. Consequently, HiCUP identifies and removes these unwanted species by checking if the forward and reverse reads of a di-tag map to the same genomic restriction fragment, but unlike circularised fragments the reads are orientated towards each other. Furthermore, HiCUP divides this category into two sub-categories depending on whether the DNA fragment end overlaps a restriction fragment cut site. If the fragment end does overlap it is termed a "dangling end" ( Figure 2d), but if it does not it is termed an "internal fragment" ( Figure 2e). Overlapping a restriction enzyme cut site is important since it is here that biotin is incorporated and consequently a high proportion of dangling ends may be indicative of failure to remove biotin residues from non-ligated DNA fragments during the Hi-C protocol "chewback" step ^13^. A high proportion of internal fragments suggests the experimental protocol may be improved in some other way: since internal fragments are derived from DNA positioned away from the restriction enzyme cut-site, these fragments should not have incorporated a biotin tag during the Hi-C ligation step. If, however, the streptavidin pull-down was not efficient, it could be assumed that a large proportion of these internal fragments arose from the genomic background. Alternatively, the presence of these internal fragments may be explained by the aberrant incorporation of biotin, possibly a result of DNA restriction digestion at non-canonical sites.

***Validating insert size.*** HiCUP places aligned di-tags on an *in silico* digested genome to calculate the theoretical length of the Hi-C insert and removes those not falling within the range set by the size-selection step of the protocol ( Figure 2g). Explanations for such discrepancies include a read being incorrectly mapped or a putative di-tag containing multiple internal fragments or dangling ends. It is also possible that sequence variation between the sample DNA and reference genome leads to the loss or creation of restriction sites in the sample material. While such events are not common, the hallmark of restriction enzyme site generation is sometimes observed in Hi-C datasets, manifesting as an aggregation of reads orientated towards the novel restriction site.

***Hi-C protocol variations.*** The Hi-C protocol may be modified by substituting sonication for a second digestion step, using a different restriction enzyme. The HiCUP pipeline includes additional filters when following the double-digest protocol, since the start of every read should now correspond to a cut site (in contrast to sonication, which is essentially a random fragmentation process). Reads not beginning exactly at a cut site are removed. Furthermore, to be considered valid a di-tag should have been generated by blunt-ended ligation between fragment ends created by the first restriction enzyme used in the protocol.

***PCR duplicates.*** Considering the huge number of theoretical pairwise interactions, it is likely that observed duplicate di-tags are the result of PCR amplification ^13^ (as demonstrated in the Results section). These duplicates will unduly strengthen the case for a given genomic interaction and therefore HiCUP removes all but one representative copy of the di-tag ( Figure 2f).

**Pipeline output:** the final valid dataset is in BAM/SAM format, with read pairs constituting a di-tag placed on adjacent lines within the file. This format is readily amenable for post-pipeline visualisation and analysis.

The relative abundance of the different classes of di-tag produced by the Hi-C assay are a direct result of the experimental protocol and therefore provide a useful QC diagnostic to identify ways in which the procedure may be improved. To enable users to benefit from these observations, HiCUP provides statistics documenting each step of the pipeline.

### Operation

HiCUP should be run on a Unix-based operating system (tested using CentOS v6.2) with Perl (tested using v5.10.1) installed. The pipeline requires a functional version of Bowtie (tested using v1.1.0) or Bowtie 2 (tested using v2.2.5) for mapping reads to a specified reference genome. Compression of SAM files to BAM format requires SAMtools (v0.1.18 or later). HiCUP may also compress or decompress files using gzip (tested using v1.3.12). For full functionality HiCUP requires the statistical programming language R (tested using v3.1.2). We recommend each file processed in parallel is allocated at least 5GB RAM.

**Figure 3.**
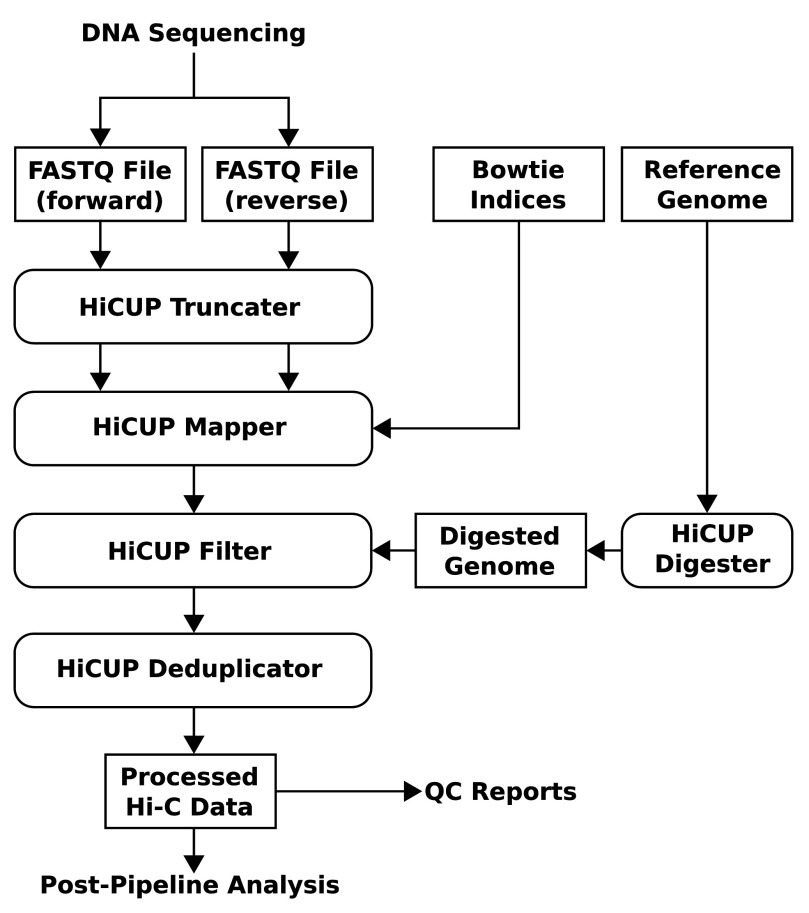
Flow diagram summarising the HiCUP pipeline. HiCUP takes FASTQ files generated by DNA sequencing and produces cleaned mapped data accompanied with QC reports. The bulk of the pipeline comprises 4 scripts: Truncater, Mapper, Filter and Deduplicator. These are executed in turn by the HiCUP master script which controls data flow through the pipeline. (The diagram uses rectangles with angled or rounded edges to represent data files or HiCUP Perl scripts respectively.)

The main body of the pipeline consists of four Perl scripts each performing a specific task. The first stage is the truncation step in which the putative Hi-C ligation junction is identified and reads are cut at this point. The second stage involves mapping reads independently and then pairing each read with its counterpart. The third stage filters out di-tags that are Hi-C artefacts, and the final de-duplication step involves removing identical di-tags. There is also a HiCUP master script that regulates data flow and executes each step of the pipeline ( Figure 3).

## Results

### Duplicate di-tags are PCR artefacts

As discussed previously, the final stage of HiCUP removes — retaining one copy — duplicate di-tags from the dataset. When considering the theoretical number of di-tags that may be generated it is reasonable to assume that exact duplicates are the result of PCR amplification and do not represent independent Hi-C ligation events. We addressed whether this assumption is valid with an experiment in which one of four barcode adapters were ligated randomly to both ends of a di-tag. We followed our standard Capture Hi-C protocol ^2^, except that all four barcode adapters were mixed at equimolar ratios in the ligation reaction. The two technical replicates named here as Sample 1 (European Nucleotide Archive accession SAMEA2421737) and Sample 2 (European Nucleotide Archive accession SAMEA2421733) were CHi-C libraries of foetal liver cells from mouse (strain C57BL/6) embryos at day 14.5 of development.

**Figure 4.**
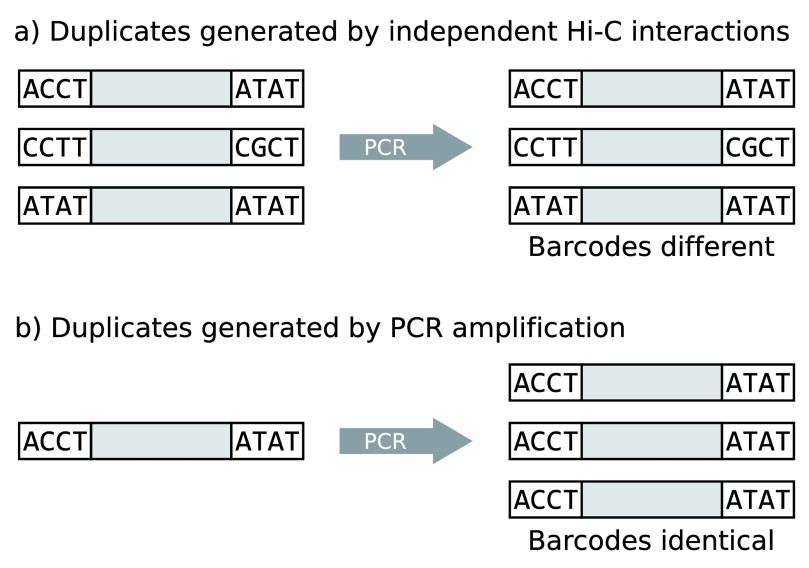
Experiment to determine whether Hi-C duplicates represent genuine independent interaction events or are the product of PCR amplification. The diagram shows di-tags (shaded rectangles) delimited by a pair of barcoded sequencing adapters.

Assuming equal numbers of each barcode, all 16 barcode-barcode permutations should occur with equal frequency. Crucially, the barcodes are incorporated into the di-tags before PCR amplification, providing a test for the origin of the duplicates: those representing independent Hi-C events are most likely to possess differing barcodes ( Figure 4a), whereas di-tags with a single common origin amplified by PCR should have identical barcodes ( Figure 4b).

The two independent libraries were sequenced and the resulting FASTQ reads were classified by barcode i.e. the first four base pairs of the polynucleotide. The reads were mapped and filtered with HiCUP, but duplicates were retained. The barcode sequences were then quantified revealing that 71.3% corresponded to a pre-defined sequence in Sample 1 and 71.5% in Sample 2. Each valid barcode was then quantified. For both samples the barcode CCTT was most prevalent (Sample 1: 9,889,602; Sample 2: 16,368,793), and for both samples the barcode CGCT was the least common (Sample 1: 2,991,820; Sample 2: 5,002,346).

Despite the deviation from the ideal and expected result in which all barcode combination frequencies were equal, it was still possible to address the source of duplicate di-tags. To achieve this, the barcode combination of each duplicate was recorded. Duplicates arising from PCR amplification should have had identical barcode combinations, in contrast to *bone fide* initial Hi-C interaction events. Table 1 shows the result of this quantification, demonstrating that the overwhelming majority of duplicate di-tags are delimited by identical barcode combinations, almost certainly a result of PCR amplification.

**Table 1.** Di-tags possessing one barcode combination. The Copies column refers to how many times a di-tag was observed in the dataset, the Di-tags column refers to the number of different di-tags observed with the specified number of copies, and the One column lists the percentage of di-tags in which all the copies possessed identical barcodes.

	Sample 1		Sample 2	
Copies	Di-tags	One(%)	Di-tags	One(%)
2	767,342	**99.4**	1,873,527	**99.6**
3	36,789	**99.0**	147,629	**99.4**
4	1,981	**98.6**	13,483	**99.3**
5	112	**96.4**	1,326	**99.1**
6	8	**100.0**	136	**100.0**
7	-	-	10	**100.0**
8	-	-	1	**100.0**

To assess the impact of retaining duplicate di-tags we detected significant interactions in HiCUP-processed CHi-C datasets using the CHiCAGO pipeline (five datasets were processed: Samples 1 and 2 are described previously and Samples 3, 4 and 5 were generated from mouse embryonic stem cells and have the Gene Expression Omnibus accession GSM1888519). The analysis showed that removing duplicate di-tags substantially reduced the number of called significant interactions (see Table 2). This was not surprising owing to the vast number of theoretical interactions, meaning that fragment-fragment contacts repeated only a small number of times were likely to be statistically significant.

**Table 2.** Results of analysing HiCUP-processed CHi-C data with CHiCAGO. The table shows the number of statistically significant interactions identified in each of the datasets when duplicate di-tags are retained (Dups) or removed (No dups). The percentage of unique di-tags in each dataset is also shown. (*Unique corresponds to the percentage of di-tags retained after removing all but one representative copy of each di-tag.)

		Significant interactions	
Sample	Unique(%)*	Dups	No dups
1	95.2	166,255	49,365
2	92.3	328,336	69,394
3	13.8	3,830,718	12,419
4	17.5	2,931,635	5,041
5	19.2	2,988,112	3,527

Consequently, when considering that PCR results in certain di-tags being amplified disproportionately ^14^, and that only a small number of observed fragment-fragment contacts are needed to qualify an interaction as statistically significant, retaining PCR duplicates will lead to an erroneous interpretation of the data. Furthermore, this over-calling of significant interactions becomes more problematic as the proportion of duplicate di-tags increases.

## Use cases

HiCUP was used to process several Hi-C datasets available on public repositories. Table 3 summarises the results. The bacterial sample was a species of *Caulobacter crescentus* (SRR824843) ^15^; human refers to *Homo sapiens* (SRR027963) ^1^; fruit fly refers to *Drosophila melanogaster* (SRR389762) ^16^ and yeast was a strain of *Schizosaccharomyces pombe* (SRR1271321) ^17^.

**Table 3.** Summary results of processing previously published datasets with HiCUP. Each value is represented as a percentage of the reads or read-pairs processed at the given stage of the pipeline. (*Unique corresponds to the percentage of di-tags retained after removing all but one representative copy of each di-tag.)

Sample	Bacteria	Human	Fruit Fly	Yeast
Truncated	7.9	7.2	0.0	8.9
Unique map Multi-map Paired Valid pairs Circularised Dangling ends Same internal Re-ligation Contiguous Wrong size	93.3 1.2 84.1 73.2 0.9 6.7 12.2 5.7 1.1 0.2	59.3 11.7 33.4 65.2 0.8 3.8 8.6 1.7 0.1 19.9	63.8 27.4 54.4 34.8 0.2 5.5 26.5 17.9 12.2 2.9	80.6 4.0 68.1 17.1 3.7 17.1 56.6 2.1 0.1 3.4
Unique* Trans	64.1 0.0	99.6 53.9	61.5 21.1	98.4 15.1

The table shows key statistics produced by HiCUP when processing Hi-C datasets. All the samples except Fruit Fly (in which the protocol did not include the fill-in/blunt-ended ligation step) show an appreciable percentage of truncated reads, meaning that the Hi-C ligation junction sequence was identified. Failure to detect the ligation junction sequence after following a standard Hi-C protocol provides a first indication that the Hi-C library is of poor quality. The samples mapped with variable efficiency to their respective reference genomes and the percentage of valid di-tags also varied considerably, but the yeast sample is particularly noticeable in returning a high proportion of internal fragments. This may be the result of additional technical difficulties in generating Hi-C libraries from yeast cells, requiring certain areas of the protocol to be further refined for reasons discussed previously.

## Discussion

HiCUP is software tailored for processing Hi-C data and has been publicly available for several years. HiCUP maps sequence data in a manner optimised for the Hi-C protocol and then removes commonly encountered experimental artefacts which could otherwise lead to incorrect inferences being made about the conformation of a genome. Furthermore, HiCUP provides statistics summarising each stage of the pipeline which may help in refining the experimental protocol. For example, a high proportion of circularised di-tags suggests inadequate cross-linking, whereas a high proportion of dangling ends implies the chewback step to remove biotin from unligated restriction fragments was inefficient ^13^. The summary statistics also include the proportion of *trans* (inter-chromosomal) interactions to *cis* (intra-chromosomal) interactions. A high *trans/cis* ratio is indicative of a poor library, since spurious ligation events will tend to be between genomic loci on different chromosomes ^18^.

HiCUP is flexible, allowing the user to specify numerous parameters and the final output is in the commonly used BAM/SAM format: which is compatible with, or may be converted to a format compatible with a myriad of analysis tools. Indeed, HiCUP has already been used to process and analyse Hi-C and CHi-C data in conjunction with GOTHiC ^2– 4^, Homer ^18, 19^ and Hicpipe ^18^. One recent study used HiCUP-processed Hi-C data to shed light on how GWAS SNPs found in genome deserts may modulate gene activity ^20^. Another publication characterised architectural changes in genome organisation in age-related cellular senescence ^19^. HiCUP was also used in a study to compare the efficiency of two different Hi-C protocols ^18^. Finally, four other research articles pioneered targeted Hi-C capture techniques, enriching for gene promoter-containing fragments to elucidate regulatory networks in mice ^2, 4, 21^ and humans ^3^. In addition to these studies, HiCUP is being used to process data in numerous ongoing projects and is under active development to meet the demands of the burgeoning fields of Hi-C and CHi-C in furthering our understanding of three-dimensional genomic structure, organisation and regulation.

## Software availability

1. URL link to where the software can be downloaded from or used by a non-coder: www.bioinformatics.babraham.ac.uk/projects/hicup
2. URL link to the author’s version control system repository containing the source code: www.bioinformatics.babraham.ac.uk/projects/hicup
3. Archived source code as at time of publication: http://dx.doi.org/10.5281/zenodo.33388 ^22^
4. Software license: GNU GPL v3 or later.
