# Supplementary material for: HiCUP: pipeline for mapping and processing Hi-C data
Source: F1000Res. 2015 Nov 20;4:1310. [Version 1] doi: 10.12688/f1000research.7334.1 (PMC4706059; doi:10.12688/f1000research.7334.1)
Supplement: Peer review history

# Peer review: Referee response for version 1

**DOI:** 10.5256/f1000research.7903.r11295

**Published:** 2015-12-07

**Authors:** Nicola Neretti

**Affiliations:** 1 Department of Molecular Biology, Cell Biology and Biochemistry, Division of Biology and Medicine, Institute for Brain and Neural Systems, Brown University, Providence, RI, USA

The authors describe a novel pipeline for mapping and littering of Hi-C data and demonstrate evidence that duplicate di-tags are PCR artifacts. This pipeline has already been successfully used in several studies. Following is a list of questions and comments that should be addressed by the authors to improve the clarity of their manuscript.

1. How does the pipeline handle the case of restriction sites that are very close to the beginning of a read? Is there a minimum size for a truncated read to be included in the analysis?
2. The “Relegation of adjacent restriction fragments” section is not completely clear to me. Fragment nr. 4 in Figure 2b could be quite large and removing fragments which include 2 restriction sizes  might remove true events corresponding to local looping of the DNA. Is there any experimental evidence that such fragments are predominantly artifacts of re-ligation or incomplete digestion and should be excluded? Or are the authors using the theoretical length of the Hi-C insert to inform this type of filtering? A more explicit description of size-selection would be beneficial here.
3. The authors present evidence that duplicate di-tags are PCR artifacts. This is in true for the standard Hi-C protocol, which from what I can see, was used to generate the datasets described in the result section. Because the HiCUP pipeline has been developed to handle the double-digestion protocol as well, could the author comment about duplicate di-tags and PCR artifacts in this context? With a double digestion protocol, the probability of truly obtaining the same fragment multiple times is much higher because the DNA is cut at fixed locations (as opposed to randomly via sonication).
4. The pipeline removes fragments with insert size less than expected by size selection. Structural variation (e.g. deletions) could yield di-tags with larger than expected theoretical insert size. Such di-tags would be valid and informative. Either an exploration or discussion of how structural variation affects this filtering step would be be helpful.
5. The percentages reported in Table 3 are difficult to interpret and compare. They clearly do not sum to 100%, so the authors should provide a more detailed description of what each percentage corresponds to (e.g. % of total reads or % of total mapping reads, etc.).

I have read this submission. I believe that I have an appropriate level of expertise to confirm that it is of an acceptable scientific standard, however I have significant reservations, as outlined above.

# Peer review: Referee response for version 1

**DOI:** 10.5256/f1000research.7903.r11292

**Published:** 2015-12-07

**Authors:** Juan M. Vaquerizas, Kruse Kai

**Affiliations:** 1 Max Planck Institute for Molecular Biomedicine, Muenster, Germany

This manuscript by Wingett and collaborators describes HiCUP, a pre-processing pipeline for Hi-C data. The pipeline is designed as an executable command-line tool that includes the most common pre-processing steps for Hi-C datasets, including read truncation and mapping, filtering, assignment of read to restriction fragments, detection and removal of PCR duplicates and generation of quality evaluation plots. The pipeline is solid and well implemented. We have downloaded and tested the current version of the pipeline using one of our locally produced Hi-C datasets, and the filtering results of the pipeline are equivalent to our locally implemented pre-processing pipelines. Therefore, we are happy with the implementation of the software and have no major concerns with the manuscript.

We only have a few minor comments that we hope will help the authors to improve the software.

1. Table 1
  It would be useful to include a further column that would display the % calculated against the total number of di-tags per sample. This would allow the user to quickly determine the overall amount of PCR-duplicates included in the library.
2. HTML reporting
  Given that the tool is aimed to provide the user with an easy way to highlight errors with Hi-C libraries, it would be useful to implement the following in the HTML report:
  - include a clearer description of how the cutoff for reads that are “too short to map” is chosen, and indicate if this can be changed by the user
  - include labelling to indicate what part of the reporting corresponds to the “read” level and what corresponds to the “pairs” level.
  - include a plot with the inwards/outwards bias as function of distance from restriction fragment as proposed by Jin *et al.*, (2013) ^1^. This is already calculated by the pipeline, but the representation of the data will help the user to determine whether the library has specific issues with under-digestions or ligation artefacts.
3. Screenshots
  The manuscript could include some screenshots of the reporting output to guide the user through it and to highlight what are the key indicators of good/bad quality datasets (some of these could even be obtained using simulated datasets with specific biases). This can also be provided as a guided example in the online documentation of the pipeline.
4. Ratio trans/cis interactions
  The manuscript states that “A high trans/cis ratio is indicative of a poor library, since spurious ligation events will tend to be between genomic loci on different chromosomes [ref 18]”.
  While we agree that this is a possible interpretation of that scenario, it should be noted that the trans/cis ratio depends on the genome’s size and the number of chromosomes that each species has, and it might also be related with specific higher-order chromatin conformations, or depend on the specific Hi-C protocol used in that experiment. Therefore, the authors might not want to generalise the interpretation of the high trans/cis ratio, or, at least, give more specific guidance regarding the interpretation of this ratio. For example, the four datasets included in this manuscript would produce trans/cis ratios of 0, 0.18, 0.52 and 1.18 for bacteria, yeast, fly and human, respectively, which in this case correlate with the genome size and the number of chromosomes in each species, but that are not necessarily indicative of the quality of these datasets.
5. Implementation and code availability
  Run on a sample of one of our locally produced Hi-C data sets (12 million paired reads), the HiCUP pipeline finishes in a reasonable amount of time (~2 hours with sequential processing of two files, a little more than half the time when it is run on two threads [2300 Mhz AMD Opteron Processor, 64Gb RAM]).
  The configuration file could be very convenient, but required some copy-pasting of file paths. Regarding this, it is unclear why R can be automatically detected, but the full path of bowtie2 has to be specified. In addition, it might be convenient to also add the HiCUP_digester step of the pipeline to the main HiCUP executable
  The link to the software package was identical to the version control system link, and it was not straightforward to find the link to the self-hosted bug report system. We think that having the ability to review code online, file bug reports, and contribute to the package using a more generally used online repository (such as GitHub) would make HiCUP even more useful.

We have read this submission. We believe that we have an appropriate level of expertise to confirm that it is of an acceptable scientific standard.

## References

[1] JinFLiYDixonJRSelvarajSYeZLeeAYYenCASchmittADEspinozaCARenB: A high-resolution map of the three-dimensional chromatin interactome in human cells. *Nature*.2013;503(7475) : 10.1038/nature12644 290-4 10.1038/nature12644 24141950PMC3838900

# Peer review: Referee response for version 1

**DOI:** 10.5256/f1000research.7903.r11293

**Published:** 2015-12-02

**Authors:** Ferhat Ay

**Affiliations:** 1 Feinberg School of Medicine, Northwestern University, Chicago, IL, USA

This article describes the HiCUP pipeline that has already been used by many for mapping and initial processing of Hi-C data. HiCUP is a useful tool for processing both traditional Hi-C data and more recent Capture Hi-C data. The article clearly describes what HiCUP does and what it does not. It also provides a nice study on the origin of duplicated di-tags showing that nearly all such duplicates are due to PCR amplification. Other than my few comments below, I am happy with the article and want to note that I have successfully downloaded and used HiCUP pipeline to process number of different Hi-C data sets.

- Even though mentioned as out of scope, it would still be useful to have a simple normalization method implemented with HiCUP. Maybe as simple as "vanilla coverage normalization" mentioned in Rao *et al. *which is a single step of matrix balancing.
- Another useful feature would be to have HiCUP generate an HTML report that basically links together all different figures created by the pipeline. I wrote such scripts to generate an HTML for my local HiCUP installation.
- "Fit-Hi-C takes renormalized data to identify mid-range intra-chromosomal interactions" --> "Fit-Hi-C takes normalized data to identify statistically significant intra-chromosomal interactions".
- If possible, modify Figure 2F to reflect that one copy of the duplicates is valid and others are discarded.
- Sequence variation between sample and the reference can be in the form of copy number changes or other aberrations. It should be noted that HiCUP does assume these do not happen.
- In "Operation", put the names of scripts in parentheses where they are described.
- In Figure 3, indicate in which steps the restriction cut site information is used (e.g. truncater, digester, filter).
- In "Results", mention explicitly that they hold for the analyzed case where sonication is the choice instead of a second digestion with a restriction enzyme. It may be the case that a substantial part of duplicates are not PCR related for the latter case where the theoretical number of di-tags is limited compared to sonication.
- In Table 3, use horizontal lines to demarcate where one step ends and the other starts. Otherwise, it is difficult to understand what each percentage is out of.

I have read this submission. I believe that I have an appropriate level of expertise to confirm that it is of an acceptable scientific standard.

## References

[1] RaoSSHuntleyMHDurandNCStamenovaEKBochkovIDRobinsonJTSanbornALMacholIOmerADLanderESAidenEL: A 3D map of the human genome at kilobase resolution reveals principles of chromatin looping. *Cell*.2014;159(7) : 10.1016/j.cell.2014.11.021 1665-80 10.1016/j.cell.2014.11.021 25497547PMC5635824
